# Delayed Postoperative Rehabilitation and Physical Function Recovery Following Late Open Conversion After Endovascular Aneurysm Repair

**DOI:** 10.7759/cureus.114515

**Published:** 2026-08-13

**Authors:** Yuki Yoshimura, Ayaka Ueki, Yukihiro Miyagawa

**Affiliations:** 1 Department of Rehabilitation, Kokura Memorial Hospital, Kitakyushu City, JPN

**Keywords:** endovascular aneurysm repair, late open conversion, open surgical repair, physical function, rehabilitation

## Abstract

Background

Late open conversion (LOC) after endovascular aneurysm repair (EVAR) is a highly invasive and technically complex procedure. However, postoperative rehabilitation progress and changes in physical function following LOC have not been sufficiently investigated. This study aimed to compare postoperative rehabilitation progress and changes in physical function between patients undergoing LOC after EVAR and those undergoing primary open surgical repair (OSR).

Methods

This single-center retrospective cohort study included 63 patients who underwent elective primary OSR or LOC between December 2022 and December 2025. The primary outcome was postoperative rehabilitation progress, assessed by the days to first ambulation and independent 100-m walking. Secondary outcomes were changes in physical function, including the Short Physical Performance Battery (SPPB) and six-minute walk distance (6MWD), from before surgery to hospital discharge. Changes in physical function were analyzed using two-way repeated-measures analysis of variance.

Results

The study included 46 patients in the primary OSR group and 17 in the LOC group. The LOC group required significantly more days to achieve first ambulation (5 (2-8) vs. 2 (2-2) days, p < 0.001) and independent 100-m walking (8 (6-13) vs. 5 (4-6) days, p < 0.001). Significant group-by-time interactions were observed for the SPPB (p = 0.004) and 6MWD (p = 0.048). At hospital discharge, the LOC group had lower SPPB scores (10.2 ± 1.8 vs. 11.4 ± 1.0 points, p = 0.001) and shorter 6MWD (288.9 ± 87.0 vs. 372.2 ± 97.9 m, p = 0.003) than the primary OSR group.

Conclusions

Compared with patients undergoing primary OSR, those undergoing LOC after EVAR experienced delayed postoperative rehabilitation and greater deterioration in physical function, particularly in lower-extremity physical performance and exercise capacity. These findings may reflect the combined effects of greater surgical invasiveness, procedural complexity, and differences in baseline characteristics.

## Introduction

Endovascular aneurysm repair (EVAR) has become a standard treatment for abdominal aortic aneurysm (AAA) in Japan since national reimbursement was introduced in 2007 [1]. Currently, EVAR accounts for approximately 60% of all AAA repairs performed in Japan [2]. Compared with conventional open surgical repair (OSR), EVAR is less invasive and is associated with lower perioperative mortality, fewer postoperative complications, and shorter hospital stays [3,4]. Consequently, EVAR has become the preferred treatment option for elderly patients and those at high surgical risk [3].

Despite these advantages, EVAR is associated with unique late complications, including endoleaks, device migration, and graft infection, necessitating lifelong imaging surveillance [5]. Many patients with these complications require secondary endovascular interventions; however, when endovascular treatment is no longer feasible, late open conversion (LOC) is required [6]. The reported incidence of LOC ranges from 1.5% to 4.3% after EVAR, with endoleaks representing the most common indication [5,7,8]. Although LOC is an effective salvage procedure for preventing aneurysm rupture and controlling graft infection, it is technically more demanding and considerably more invasive than primary OSR because of severe perigraft inflammation, dense adhesions around the implanted stent graft, and the frequent need for suprarenal or supraceliac aortic cross-clamping [9].

Because LOC is substantially more invasive than primary OSR and often requires more complex perioperative management, postoperative rehabilitation may be delayed, and recovery of physical function may be impaired. However, postoperative rehabilitation progress and longitudinal changes in physical function following LOC have not been sufficiently investigated, and comparative data with patients undergoing primary OSR remain limited.

Therefore, this study aimed to compare postoperative rehabilitation progress (primary outcome) and changes in physical function (secondary outcomes) between patients undergoing LOC after EVAR and those undergoing primary OSR. We hypothesized that patients undergoing LOC would experience delayed postoperative rehabilitation and greater deterioration in physical function than those undergoing primary OSR.

## Materials and methods

Study design and participants

This single-center retrospective cohort study was conducted using data extracted from electronic medical records. Consecutive patients who underwent elective primary OSR or LOC at our institution between December 2022 and December 2025 were screened for eligibility. Patients were excluded if they (1) experienced postoperative complications classified as Clavien-Dindo grade III or higher [10], (2) had pre-existing musculoskeletal or cerebrovascular disorders that could affect physical function assessment, or (3) refused postoperative rehabilitation.

The study was conducted in accordance with the Declaration of Helsinki. Patient information was anonymized before analysis, and an opt-out approach was implemented through the institutional website to provide patients with the opportunity to decline participation. The study protocol was approved by the Institutional Review Board of our hospital (Approval No. 26070201).

Outcome measures

The primary outcome was postoperative rehabilitation progress, assessed by the days to first ambulation and the days to independent 100-m walking. Secondary outcomes were changes in physical function from preoperative assessment to pre-discharge assessment, assessed using the Short Physical Performance Battery (SPPB), six-minute walk distance (6MWD), handgrip strength, arm muscle circumference (AMC), and calf circumference (CC). Intensive care unit (ICU) length of stay and postoperative hospital length of stay were also recorded as perioperative clinical outcomes.

Data collection

Demographic variables included age, sex, body mass index (BMI), and the Barthel Index (BI) at admission. Comorbidities included hypertension, dyslipidemia, diabetes mellitus, cerebrovascular disease, coronary artery disease, chronic respiratory disease, and chronic musculoskeletal disorders.

For patients undergoing LOC, the type of endoleak and the interval from primary EVAR to LOC were also recorded.

Laboratory variables included serum albumin, serum creatinine, estimated glomerular filtration rate (eGFR), and hemoglobin. Cardiopulmonary assessments included left ventricular ejection fraction (LVEF), percent predicted vital capacity (%VC), and forced expiratory volume in one second (FEV1.0%).

Operative variables included anesthesia time, operative time, intraoperative blood loss, transfusion volume, postoperative fluid balance normalized to preoperative body weight (POFB/PBW, %), and duration of endotracheal intubation. POFB/PBW was calculated as postoperative fluid balance divided by preoperative body weight multiplied by 100 and was used as an indicator of perioperative fluid management [11].

Physical function assessment

Physical function was assessed on the day before surgery and on the day before hospital discharge using the SPPB, 6MWD, handgrip strength, AMC, and CC. All physical function assessments were performed by two physical therapists, each with more than 10 years of clinical experience. The assessors were not blinded to the type of surgical procedure. The SPPB consists of three components: standing balance, a 4-m gait speed test, and the five-times chair stand test, with a total score ranging from 0 to 12 points [12]. The 6MWD was assessed according to the American Thoracic Society guidelines in a 30-m corridor [13]. Handgrip strength was measured using a Smedley-type dynamometer. Measurements were performed twice for each hand with the participant in the standing position and the arms hanging naturally at the sides. The average of the highest value obtained from each hand was normalized by BMI to account for differences in body size [14]. AMC was calculated using arm circumference and triceps skinfold thickness according to a previously described method [15]. Calf circumference was measured at the widest portion of the lower leg [15]. All anthropometric measurements were performed three times, and the mean value was used for analysis.

Postoperative rehabilitation program

Postoperative rehabilitation was performed in accordance with the 2021 Japanese Circulation Society/Japanese Association of Cardiac Rehabilitation Guidelines for Rehabilitation in Patients With Cardiovascular Disease [16]. Physical therapists initiated rehabilitation on postoperative day 1 according to each patient's clinical condition. Rehabilitation progressed from sitting and standing to ambulation, followed by progressive walking training, activities of daily living (ADL) training, aerobic exercise using a cycle ergometer or treadmill, and resistance exercises using body weight. Rehabilitation sessions were conducted five days per week, with each session lasting approximately 30-40 minutes, according to each patient's clinical condition.

Statistical analysis

Continuous variables are presented as the mean ± standard deviation or median with interquartile range, whereas categorical variables are presented as frequencies and percentages. The normality of continuous variables was assessed using the Shapiro-Wilk test. Between-group comparisons were performed using the unpaired Student's t-test or Mann-Whitney U test, as appropriate, and categorical variables were compared using Fisher's exact test. Changes in physical function before surgery and before hospital discharge were analyzed using two-way repeated-measures analysis of variance (group × time). When a significant interaction was observed, paired t-tests were performed to compare preoperative and postoperative values within each group, and unpaired Student's t-tests were used for between-group comparisons at hospital discharge. No missing data were present among the variables included in the analyses. All statistical analyses were performed using R version 4.2.2 (R Foundation for Statistical Computing, Vienna, Austria). A two-sided P value < 0.05 was considered statistically significant. Because this was a retrospective cohort study, an a priori sample size calculation was not performed. The sample size was determined by the number of eligible patients who met the inclusion criteria during the study period.

## Results

Primary Outcome: Postoperative Rehabilitation Progress

For the primary outcome, patients in the LOC group required significantly more days to achieve first ambulation (5 (2-8) vs. 2 (2-2) days, p < 0.001) and independent 100-m walking (8 (6-13) vs. 5 (4-6) days, p < 0.001) than those in the primary OSR group. ICU length of stay (3 (1-7) vs. 1 (1-1) days, p < 0.001) and postoperative hospital length of stay (19 (15-23) vs. 14 (13-16) days, p < 0.001) were also significantly longer in the LOC group.

Secondary Outcomes: Changes in Physical Function

**Figure 1:**
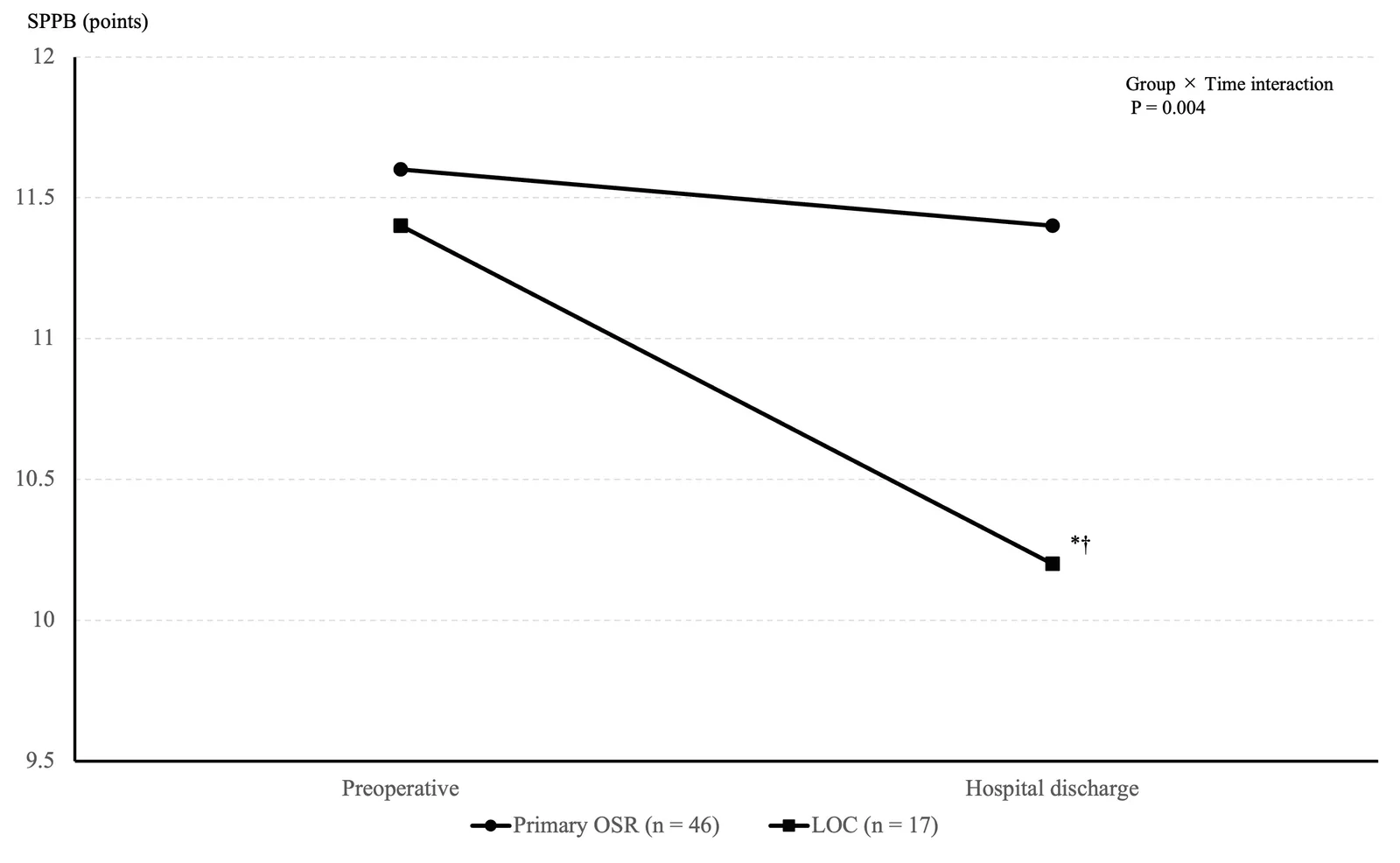
Changes in Short Physical Performance Battery (SPPB) scores from before surgery to hospital discharge. Changes in Short Physical Performance Battery (SPPB) scores in patients undergoing primary open surgical repair (Primary OSR) and late open conversion (LOC) after endovascular aneurysm repair. Data are presented as mean values. Group × time interaction was analyzed using two-way repeated-measures analysis of variance. *: p < 0.05 versus preoperative value. †: p < 0.05 versus the primary open surgery group at hospital discharge.

**Figure 2:**
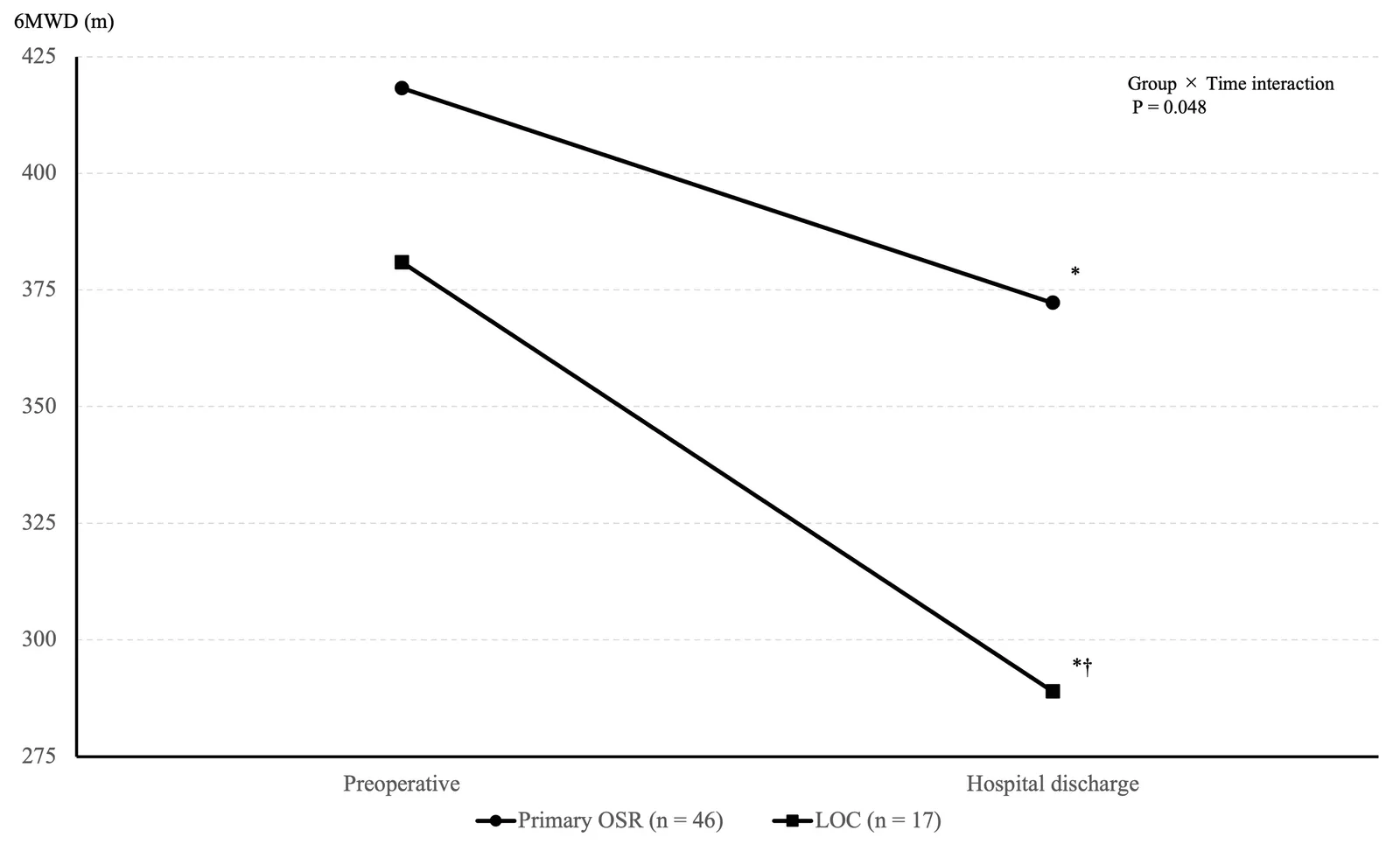
Changes in six-minute walk distance (6MWD) from before surgery to hospital discharge. Changes in six-minute walk distance (6MWD) in patients undergoing primary open surgical repair (Primary OSR) and late open conversion (LOC) after endovascular aneurysm repair. Data are presented as mean values. Group × time interaction was analyzed using two-way repeated-measures analysis of variance. *: p < 0.05 versus preoperative value. †: p < 0.05 versus the primary open surgery group at hospital discharge.

Changes in physical function are summarized in Table 2. Significant group-by-time interactions were observed for the SPPB score (p = 0.004; Figure 1) and 6MWD (p = 0.048; Figure 2). Post hoc analyses demonstrated that the SPPB score significantly decreased from 11.4 ± 0.7 to 10.2 ± 1.8 points in the LOC group, whereas no significant change was observed in the primary OSR group (11.6 ± 0.7 to 11.4 ± 1.0 points). The 6MWD significantly decreased in both the primary OSR group (418.2 ± 82.1 to 372.2 ± 97.9 m) and the LOC group (380.9 ± 61.5 to 288.9 ± 87.0 m). At hospital discharge, patients in the LOC group had significantly lower SPPB scores (10.2 ± 1.8 vs. 11.4 ± 1.0 points, p = 0.001) and shorter 6MWD (288.9 ± 87.0 vs. 372.2 ± 97.9 m, p = 0.003) than those in the primary OSR group.

Baseline and Perioperative Characteristics

Baseline and perioperative characteristics are summarized in Table 1. Compared with patients in the primary OSR group, those in the LOC group were significantly older (78.0 ± 3.7 vs. 73.0 ± 7.8 years, p = 0.015), had significantly higher serum creatinine levels (1.12 (0.99-1.28) vs. 0.90 (0.76-1.05) mg/dL, p = 0.006), and had significantly lower eGFR (50.4 ± 12.8 vs. 61.8 ± 16.3 mL/min/1.73 m², p = 0.011). No significant between-group differences were observed in preoperative physical function, including SPPB, 6MWD, relative grip strength, AMC, and CC.

Compared with the primary OSR group, the LOC group had significantly longer anesthesia time (433 ± 56 vs. 382 ± 69 minutes, p = 0.009) and operative time (339 ± 59 vs. 283 ± 63 minutes, p = 0.002), greater intraoperative blood loss (390 (270-750) vs. 190 (110-297) mL, p < 0.001), larger transfusion volume (2,549 (2,030-6,716) vs. 645 (479-949) mL, p < 0.001), higher POFB/PBW (11.4 (9.4-15.7)% vs. 6.6 (5.2-8.3)%, p < 0.001), and longer duration of endotracheal intubation (465 (393-5,756) vs. 355 (307-398) minutes, p < 0.001).

**Table 1:** Baseline and Perioperative Characteristics Values are presented as mean ± standard deviation, median (interquartile range), or number (%). Continuous variables were compared using the unpaired Student's t-test or Mann–Whitney U test, as appropriate. Categorical variables were compared using Fisher's exact test. OSR: Open Surgical Repair, LOC: Late Open Conversion, BMI: body mass index, EVAR: endovascular aneurysm repair, BI: Barthel Index, Alb: serum albumin, Cre: serum creatinine, eGFR: estimated glomerular filtration rate, Hb: hemoglobin, LVEF: left ventricular ejection fraction, %VC: percent predicted vital capacity, FEV1.0: forced expiratory volume in one second, POFB: postoperative fluid balance, PBW: preoperative body weight, ICU: intensive care unit.

	Overall	Primary OSR group	LOC group	Test statistic	P value
	(N = 63)	(n = 46)	(n = 17)		
Demographic characteristics					
Age, years	74.3±7.3	73.0±7.8	78.0±3.7	t = -2.50	0.015
Male sex, n (%)	56 (88)	39 (84)	17 (100)	-	0.175
BMI, kg/㎡	24.2±2.9	23.9±2.9	25.1±3.0	t = -1.47	0.147
Type of endoleak					
Type I, n (%)		-	1 (5)		-
Type II, n (%)		-	12 (70)		-
Type Ⅲ, n (%)		-	2 (15)		-
Type Ⅳ, n (%)		-	1 (5)		-
Unknown, n (%)		-	1 (5)		-
Interval between EVAR and LOC, months		-	79.5±32.1		-
Admission BI, points	100 (100–100)	100 (100–100)	100 (100–100)	W = 391	1
Comorbidities					
Hypertension, n (%)	50 (79)	38 (82)	12 (70)	-	0.311
Dyslipidemia, n (%)	36 (57)	28 (60)	8 (47)	-	0.395
Diabetes mellitus, n (%)	11 (17)	10 (21)	1 (5)	-	0.262
Cerebrovascular disease, n (%)	7 (11)	4 (8)	3 (17)	-	0.375
Coronary artery disease, n (%)	26 (41)	20 (43)	6 (35)	-	0.774
Chronic respiratory disease, n (%)	7 (11)	5 (11)	2 (11)	-	1
Chronic musculoskeletal disorders, n (%)	13 (20)	8 (17)	5 (29)	-	0.311
Laboratory data					
Alb, g/dL	4.0 (3.7–4.2)	4.0 (3.7–4.2)	4.0 (3.8–4.3)	W = 401	0.883
Cre, mg/dL	0.94 (0.79–1.12)	0.90 (0.76–1.05)	1.12 (0.99–1.28)	W = 214	0.006
eGFR, mL/min/1.73㎡	58.7±16.1	61.8±16.3	50.4±12.8	t = 2.59	0.011
Hb, g/dL	13.5±1.5	13.7±1.5	12.9±1.3	t = 1.87	0.065
Cardiopulmonary function					
LVEF, %	62.6 (58.1–64.3)	62.7 (59.3–64.9)	59.4 (56.3–63.3)	W = 481	0.163
%VC, %	115.1±19.1	117.4±20.6	109.1±12.9	t = 1.50	0.138
FEV1.0, %	69.1±9.6	68.8±9.4	70.1±10.4	t = -0.41	0.667
Preoperative physical function					
SPPB, points	11.6±0.7	11.6±0.7	11.4±0.7	t = 1.01	0.314
6MWD, m	407.9±78.3	418.2±82.1	380.9±61.5	t = 1.69	0.094
Relative grip strength, kg/BMI	1.2±0.3	1.2±0.3	1.2±0.2	t = 0.50	0.622
AMC, cm	22.7±2.6	22.6±2.9	23.0±1.6	t = -0.53	0.592
CC, cm	34.5±3.2	34.7±3.1	34.1±3.7	t = 0.56	0.573
Operative variables					
Anesthesia time, min	396±70	382±69	433±56	t = -2.66	0.009
Operative time, min	298±66	283±63	339±59	t = -3.14	0.002
Blood loss, mL	240 (120–355)	190 (110–297)	390 (270–750)	W = 147	p < 0.001
Transfusion volume, mL	940 (530–2150)	645 (479–949)	2549 (2030–6716)	W = 27	p < 0.001
POFB/PBW, %	7.1 (5.6–9.8)	6.6 (5.2–8.3)	11.4 (9.4–15.7)	W = 131	p < 0.001
Duration of endotracheal intubation, min	371 (328–423)	355 (307–398)	465 (393–5756)	W = 142	p < 0.001
Postoperative rehabilitation outcomes					
Days to first ambulation	2 (2–4)	2 (2–2)	5 (2–8)	W = 166	p < 0.001
Days to independent 100-m walking	5 (5–6)	5 (4–6)	8 (6–13)	W = 119	p < 0.001
ICU length of stay, days	1 (1–1)	1 (1–1)	3 (1–7)	W = 192	p < 0.001
Postoperative hospital length of stay, days	14 (14–18)	14 (13–16)	19 (15–23)	W = 164	p < 0.001

**Table 2:** Changes in physical function before surgery and at hospital discharge Values are presented as mean ± standard deviation. P values for the main effects (Time and Group) and the Group × Time interaction were calculated using two-way repeated-measures analysis of variance. When a significant interaction was observed, paired t-tests were performed to compare preoperative and hospital discharge values within each group, and unpaired Student's t-tests were used for between-group comparisons at hospital discharge. *: p < 0.05 versus preoperative value. †: p < 0.05 versus the primary open surgery group at hospital discharge. OSR: Open Surgical Repair; LOC: Late Open Conversion; SPPB: Short Physical Performance Battery; 6MWD: six-minute walk distance; BMI: body mass index; AMC: arm muscle circumference; CC: calf circumference.

Variable	Preoperative	Hospital discharge	Group		Time		Group × Time	
			F	p-value	F	p-value	F	p-value
SPPB (points)			8.053	0.006	17.446	p<0.001	8.914	0.004
Primary OSR group	11.6±0.7	11.4±1.0						
LOC group	11.4±0.7	10.2±1.8*†						
6MWD (m)			7.175	0.009	41.011	p<0.001	4.066	0.048
Primary OSR group	418.2±82.1	372.2±97.9*						
LOC group	380.9±61.5	288.9±87.0*†						
Relative grip strength (kg/BMI)			0.328	0.568	9.328	p<0.001	0.647	0.424
Primary OSR group	1.2±0.3	1.2±0.4						
LOC group	1.2±0.2	1.2±0.2						
AMC (cm)			0.283	0.596	4.302	0.042	2.279	0.136
Primary OSR group	22.6±2.9	21.8±2.7						
LOC group	23.0±1.6	22.0±1.6						
CC (cm)			2.464	0.121	3.875	0.054	0.791	0.380
Primary OSR group	34.7±3.1	32.7±3.0						
LOC group	34.1±3.7	31.6±3.1						

## Discussion

The present study compared postoperative rehabilitation progress and changes in physical function between patients undergoing LOC after EVAR and those undergoing primary open abdominal aortic repair. The main findings were that patients undergoing LOC experienced delayed postoperative rehabilitation and greater deterioration in lower-extremity physical performance and exercise capacity, as reflected by changes in the SPPB and 6MWD, respectively. However, these differences should not be attributed solely to LOC because differences in baseline characteristics, particularly age and renal function, may also have influenced postoperative recovery.

Early mobilization after cardiovascular surgery is recommended to prevent postoperative complications and promote functional recovery. According to the Japanese Circulation Society/Japanese Association of Cardiac Rehabilitation Guidelines, standing and ambulation are initiated on the first postoperative day, with independent walking generally expected by postoperative day 4 [16]. In the present study, patients in the LOC group required a median of 5 days to achieve initial ambulation and 8 days to achieve independent 100-m walking, compared with 2 and 5 days, respectively, in the primary OSR group. Thus, postoperative rehabilitation progress in the LOC group was delayed not only relative to the primary OSR group but also relative to the rehabilitation timeline recommended in the guidelines.

Several factors have been reported to delay postoperative rehabilitation after cardiovascular surgery, including advanced age, impaired renal function, prolonged operative time, greater surgical invasiveness, excessive blood loss, and prolonged mechanical ventilation [17-20]. Consistent with these previous reports, patients in the LOC group in the present study were older, had poorer renal function, underwent longer operations, experienced greater blood loss, required larger transfusion volumes, and remained intubated for longer periods. Because LOC is technically more demanding than primary open repair owing to severe perigraft inflammation, dense adhesions, and the frequent need for complex aortic reconstruction, greater surgical stress is often unavoidable [9]. Moreover, excessive inflammatory responses and perioperative fluid overload have been associated with delayed recovery of cardiopulmonary function following major surgery [21-24]. These perioperative factors may have contributed to delayed postoperative stabilization and rehabilitation in the LOC group. However, the LOC group was also significantly older and had poorer preoperative renal function than the primary OSR group. Therefore, the observed delay in postoperative rehabilitation may reflect the combined influence of greater surgical invasiveness and differences in baseline patient characteristics rather than the effect of LOC alone.

Regarding the secondary outcomes, preoperative SPPB scores and 6MWD did not differ significantly between the groups. Nevertheless, significant group-by-time interactions were observed for both outcomes, and patients in the LOC group had lower SPPB scores and shorter 6MWD at hospital discharge than those in the primary OSR group. These findings indicate that the postoperative trajectories of lower-extremity physical performance and exercise capacity differed between the groups. The SPPB assesses multiple domains of lower-extremity function, including balance, gait speed, and chair-rise ability, and has been associated with lower-extremity muscle performance [25,26]. The greater decline in SPPB and 6MWD observed in the LOC group may therefore reflect less favorable postoperative recovery of physical performance and exercise capacity. However, because this was an observational study and the groups differed in age and renal function at baseline, these differences cannot be attributed solely to the surgical procedure.

In contrast, no significant group-by-time interactions were observed for relative grip strength, arm muscle circumference, or calf circumference. Thus, the changes observed in SPPB and 6MWD were not accompanied by corresponding between-group differences in relative grip strength or anthropometric measures. These findings suggest that assessment of postoperative recovery after LOC should include performance-based measures such as the SPPB and 6MWD in addition to simple measurements of muscle strength and body dimensions. Such multidimensional assessment may help clinicians identify patients with delayed functional recovery after this highly invasive procedure.

This study has several limitations. First, this was a single-center retrospective study with a relatively small sample size, which may have introduced selection bias, limited statistical power, and reduced the generalizability of the findings. Second, patients who developed postoperative complications classified as Clavien-Dindo grade III or higher were excluded. Because LOC is associated with greater surgical complexity, the exclusion of patients with severe postoperative complications may have resulted in underestimation of the overall impact of LOC on postoperative recovery. Third, significant differences in age and renal function were observed between the groups, and these potential confounders were not adjusted for in the analysis. Therefore, the observed differences in postoperative rehabilitation progress and physical function cannot be attributed solely to LOC. Fourth, potentially relevant postoperative factors were not comprehensively assessed. In particular, postoperative delirium was not evaluated, although delirium has been reported to be independently associated with functional decline after cardiac surgery [27]. Thus, unmeasured postoperative factors may have influenced functional recovery. In addition, the assessors were not blinded to the type of surgical procedure, which may have introduced measurement bias. Fifth, although postoperative rehabilitation was performed according to current clinical guidelines [16] and was generally provided five days per week for approximately 30-40 minutes per session, the content and progression of rehabilitation were adjusted according to each patient's clinical condition and were therefore not completely standardized. Finally, physical function was assessed only until hospital discharge, and no post-discharge follow-up was performed. Therefore, it remains unclear whether the observed decline in physical function persisted after discharge or was associated with longer-term clinical outcomes. Future multicenter prospective studies with larger sample sizes, adjustment for potential confounders, standardized rehabilitation protocols, and long-term follow-up are warranted to confirm these findings.

## Conclusions

Compared with patients undergoing primary OSR, those undergoing LOC after EVAR experienced delayed postoperative rehabilitation and greater deterioration in physical function, particularly in lower-extremity physical performance and exercise capacity. However, these findings should be interpreted cautiously because differences in baseline characteristics, particularly age and renal function, may have contributed to the observed differences between the groups. The findings may therefore reflect the combined influence of greater surgical invasiveness, procedural complexity, and baseline patient characteristics rather than the effect of LOC alone. Further prospective studies with larger sample sizes and adjustment for potential confounders are warranted to identify factors associated with postoperative functional recovery following LOC and to establish optimal perioperative rehabilitation strategies.
